# Multidisciplinary team consultation for resectable Gastric Cancer: A propensity score matching analysis

**DOI:** 10.7150/jca.53365

**Published:** 2021-01-30

**Authors:** Yonghe Chen, Jun Xiang, Dan Liu, Jian Xiao, Fei Xiong, Kaikai Wei, Aihong Liu, Shi Chen, Yaxi Zhu, Xiaochun Meng, Junsheng Peng

**Affiliations:** 1 Department of Gastrointestinal Surgery, The Sixth Affiliated Hospital, Sun Yat-sen University, Guangzhou, 510655, China.; 2 Department of Laboratory Science, The Second Affiliated Hospital, Guangzhou University of Chinese Medicine, Guangzhou, 510105, China.; 3 Department of Medical Oncology, The Sixth Affiliated Hospital, Sun Yat-sen University, Guangzhou, 510655, China.; 4 Department of Radiology, The Sixth Affiliated Hospital, Sun Yat-sen University, Guangzhou, 510655, China.; 5 Department of Pathology, The Sixth Affiliated Hospital, Sun Yat-sen University, Guangzhou, 510655, China.; 6 Guangdong Institute of Gastroenterology, Guangdong Provincial Key Laboratory of Colorectal and Pelvic Floor Diseases, Guangzhou, 510655, China.

**Keywords:** multidisciplinary team consultation, gastric cancer, propensity score matching, survival, patient compliance

## Abstract

**Purpose:** Previous studies proposed that the multidisciplinary team (MDT) consultation could improve tumor staging accuracy and outcomes of patients with gastric malignancy. However, evidence-based reports remain limited. This study aimed to determine the effectiveness of MDT for tumor staging accuracy and outcomes of patients with resectable gastric cancer, and to explore the potential factors affecting its effectiveness.

**Methods:** This retrospective study enrolled 719 gastric cancer patients who underwent gastrectomy in our hospital. After propensity score matching, 378 patients were selected, including 189 in the non-MDT group and 189 in the MDT group. Data regarding baseline characteristics, staging, treatments, and survival were analyzed.

**Results:** The data showed that the staging accuracy in the MDT group and non-MDT group was comparable (53% vs 61% for T stage, 46.1% vs 35.3% for N stage, and 78.3% vs 78.7% for M stage). The MDT group had a higher proportion of preoperative chemotherapy (39.2% vs 28%, p=0.03) and laparoscopic surgery (82.5% vs 72%, p=0.02) than the non-MDT group. However, the achievement of R0 resection was similar in the two groups (93.7% vs 88.9%, p=0.73). There was no significant difference in the 1-year and 3-year overall survival rates between the two groups. Moreover, we observed poor patient compliance when the MDT recommended further examinations, radiotherapy, or chemotherapy before surgical interventions.

**Conclusion:** MDT consultation has limited effects on improving the staging accuracy and treatment outcomes including survival of patients with resectable gastric cancer. Poor patient compliance may be a factor affecting the effectiveness of MDT consultation.

## Introduction

Gastric cancer is the fifth most common malignancy in the world and the third leading cause of cancer-related deaths 1. Surgical resection is the main approach for the management of resectable gastric cancer. However, preoperative management (such as accurate staging, pathological assessment, chemotherapy intervention, and radiotherapy) is also critical for improving patient outcomes. There are still controversies about the treatment options and management of patients at different stages; and the recommended treatment plan varies from different guidelines 2, 3. Therefore, there has been a growing demand in multidisciplinary teams (MDT) integrating strategies of multiple disciplines, to ensure the treatment plans to be thorough, standardized, and effective 4.

The MDT approach can be broadly defined as an integrated team effort that aims to develop individualized treatment plans for patients through improved communication, coordination, and decision making between health care professionals 5, 6. Although it has been widely accepted as a “gold standard” in the care of cancer patients, current literature suggests that the quality and the effectiveness of the MDT approach could be varied among different types of cancers and different centers 6. Moreover, many factors derived from both healthcare providers and cancer patients may affect how well the MDT is implemented in the care of cancer patients; and efforts remained to be made to address these factors.

Previous studies proposed that MDT could improve the accuracy of tumor location and stage and could be beneficial to patient survival 7-9. However, these studies mainly focus on the methodology about organizing MDT management, and studies with detailed data or strong evidence demonstrating the impact of MDT consultation on treatment outcomes of cancer patients are still limited.

In this retrospective study, we analyzed the data of patients with resectable gastric cancer who underwent gastrectomy in the past 5 years, and compared the data between patients with and without MDT consultation before surgery, with the aim to determine the effect of MDT on tumor staging accuracy and treatment outcomes and to identify factors affecting its effectiveness.

## Methods

### Study population and data collection

**Figure 1:**
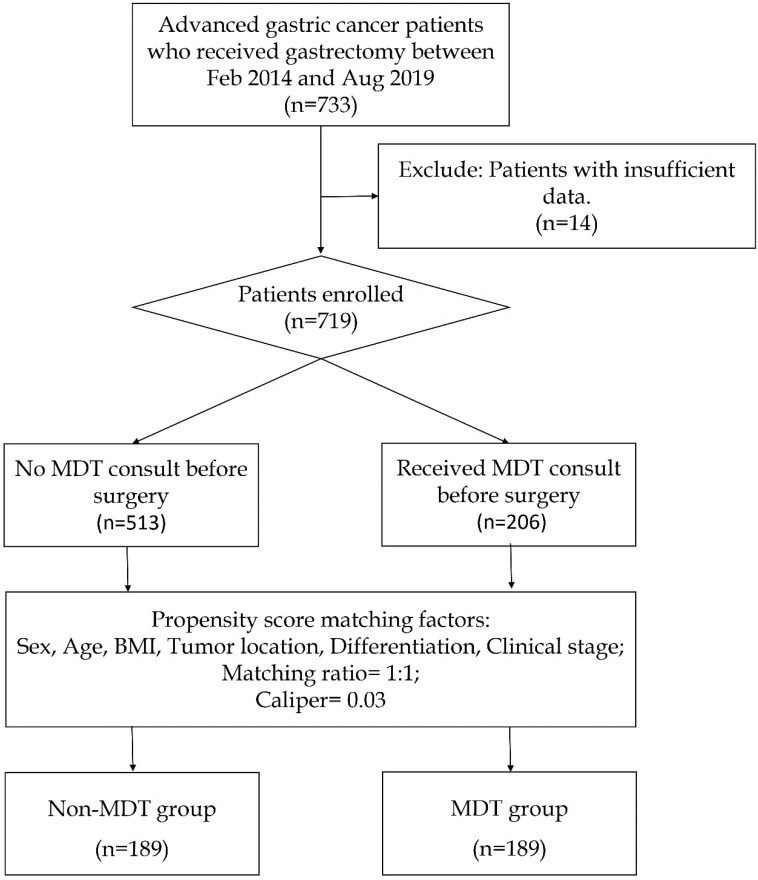
The flowchart showing the process of patient enrollment and propensity score matching.

The patients' recruitment process is shown in **Figure 1**. During the initial screening process, we identified 733 patients who underwent gastrectomy from the gastric cancer database of The Sixth Affiliated Hospital of Sun Yat-sen University from February 2014 to August 2019. Among them, 14 patients with insufficient data were excluded, leaving 719 patients, of which 206 received MDT consultation before surgery and 513 didn't. Their demographic information including gender, age, body mass index (BMI), tumor location, differentiation, clinical stage, MDT recommendation, surgical outcomes, pathological stage, and survival were collected. This study was reviewed and approved by the Ethics Committee of The Sixth Affiliated Hospital, Sun Yat-Sen University. This study was conducted in accordance with the 1964 Helsinki Declaration. All study participants, or their legal guardians, were provided with informed consent prior to study by the follow-up office.

### Multidisciplinary team consultation clinical model

The Multidisciplinary expert panel for gastric cancer at The Sixth Affiliated Hospital of Sun Yat-sen University consisted of gastroenterological surgeons, medical oncologists, radiation oncologists, radiologists, pathologists, and a coordinator. It was a weekly clinic open every Wednesday of the week. Gastric cancer patients with complicated conditions would be recommended to the MDT clinic by the doctor in charge. Generally, we required that the patients should have a confirmed diagnosis of gastric cancer with histological evidence and had complete necessary examination such as endoscopic inspection, computed tomography scan of chest, abdomen, and pelvis. On the consulting conference, the patient's history, along with the imaging and pathological slide, would be presented and reassessed by a senior radiologist and pathologist, to confirm the diagnosis and adjust the clinical stage according to the American Joint Committee on Cancer staging criteria 10.

If the T, N, M status is undetermined, the MDT would advise patients to received further inspection such as endoscopic ultrasound, positron emission tomography-computed tomography (PET-CT) or magnetic resonance imaging (MRI) to determine the tumor invasion depth, perigastric nodal status and to further differentiate suspicious metastatic site. A second conference would be held after the suggested examinations were undertaken to determine the following strategy. In cases of patients refusing these examinations, the MDT will discuss the treatment plan based on the currently available information. Generally, the MDT recommend treatment plans based on the Chinese gastric cancer diagnosis and treatment guideline 11 (Published by the National Health Commission of the PRC) and the Japanese gastric cancer treatment guidelines^12^. For patients with controversial conditions, the MDT would try to develop personalized and integrated strategies for the patients, or recruit them for clinical studies. The expert panel would explain to patients the current state of the illness and the details of recommended treatment plan. With patients' consent, the doctors in charge will arrange further examination or treatment according to the recommendation.

### Propensity score matching

To determine the impact of MDT, a propensity score matching method was used to select patients with similar baseline characteristics. To imitate randomized inclusion, matching factors included only preoperative parameters: gender, age, BMI, tumor location, differentiation, and clinical stage. The matching ratio was 1:1, the caliper was 0.03. The matched pairs were divided into MDT group and non-MDT groups. Data analysis was based on the matched cohort.

### Data analysis

Normally distributed continuous variables were expressed in the form of mean ± standard deviation and were analyzed by Student's* t*-test. Categorical variables were analyzed by the chi-square test. The survival difference between the non-MDT and MDT group was compared using the Kaplan-Meier method, and the hazard ratios were calculated in the Cox regression model, a p-value<0.05 were identified as statistically significant. All statistical analyses were performed using SPSS software ver. 25.0 (IBM, Armonk, NY, USA) and R version 4.0 software (The R Foundation for Statistical Computing, Vienna, Austria; www.r-project.org).

## Results

### Patient characteristics

The process of patient enrollment and propensity score matching was shown in **Figure 1**. Within the 719 patients enrolled, a cohort of 378 patients was selected for analysis, including 189 in the Non-MDT group and 189 in the MDT group. The patient characteristics including baseline information were shown in **Table 1**. As a result of propensity score matching, all baseline characteristics including gender, age, body mass index (BMI), tumor location and differentiation, and clinical stage were similar in these two groups. Patients were mostly male at a median age of 58 years with similar BMI. The majority of patients were diagnosed with poorly differentiated adenocarcinoma at clinical stage III. Also, the clinical stage was undetermined for one-fifth of the patients before surgery.

### Staging accuracy

The accuracy of clinical staging in both groups was listed in **Table 2**. Patients that had received preoperative chemotherapy were not included in the staging accuracy analysis, since the final pathological stage could be vastly altered by chemotherapy. The clinical stages of patients in the MDT group were all reassessed and adjusted after MDT consultation. However, the data suggested that MDT consultation did not improve the clinical staging accuracy, as the accuracy of T (MDT 53% vs Non-MDT 61%, p=0.506), N (MDT 46.1% vs Non-MDT 35.3%, p=0.26), and M stages (MDT 78.3% vs Non-MDT 78.7%, p=0.978) was all statistically similar between these two groups. The only difference we observed was that patients in the MDT group tended to be over staged for the T stage (MDT 27% vs Non-MDT 14.7%, p=0.05).

### MDT recommendations and patient compliance

As shown in **Table 3**, for all the patients at clinical stage I, MDT recommended direct surgical resection, 3 patients were recommended with endoscopic ultrasound to re-assure the T stage. For clinical stage II, most patients (70%,7/10) were recommended with direct surgical, 3 patients with bulky lymph nodes were recommended with neoadjuvant chemotherapy. For clinical stage III, the recommended strategies were divided, patients with adjacent organ invasion (T4a or T4b) or bulky lymph nodes tended to be recommended with neoadjuvant chemotherapy (39%, 45/115), otherwise direct surgery were recommended (42%, 48/115). For patients at stage IV, exploratory laparoscopy was recommended most (57%, 8/14), chemotherapy (43%, 6/14) and palliative treatment (43%, 6/14) was also frequently recommended. Patients with undetermined stage were mostly recommended with exploratory laparoscopy (56%, 22/39), subsequent radical resection or chemotherapy shall be proceeded accordingly. Patients' compliance was clearly highest with direct surgical resection, laparoscopic explorations (83%, 24/29) were accepted as a part of the resection surgery, no staging laparoscopic exploration without resection were accepted or performed. Only a minority of patients (34%, 11/32) accepted further examination. None of them accepted the recommendation of radiotherapy or palliative therapy. These data indicated that patients' compliance could play a role in affecting the effectiveness of MDT consultation.

### Treatments and clinical outcomes

As shown in **Table 1**, more patients in the MDT group received preoperative chemotherapy than in the non-MDT group (39.2% vs 28%, p=0.03). More than half of the preoperative chemotherapy regimen was modified FLOT regimen (fluorouracil plus leucovorin, oxaliplatin, and docetaxel), which was similar in both groups. Other regimens were mainly platin-based doublet regimens, such as SOX, FOLFOX or XELOX. Laparoscopic surgery was the major resection approach in both groups but was more favored in the MDT group (82.5% vs 72%, p=0.02).

As for the surgical outcome, R0 resection was successfully achieved for around 91.3% of patients; however, no statistical difference was observed between the non-MDT group and the MDT group (88.9% vs 93.7%, p=0.73). Also, the postoperative hospital stay days between these two groups were comparable (14 vs 14). Notably, 17 out of the 127 patients who received preoperative chemotherapy achieved a pathological complete response (13.4%). The other pathological stages between these two groups were also similar. It should be noted that the MDT groups harvested fewer lymph nodes than the non-MDT group (p=0.009).

### Survival analysis

**Figure 2:**
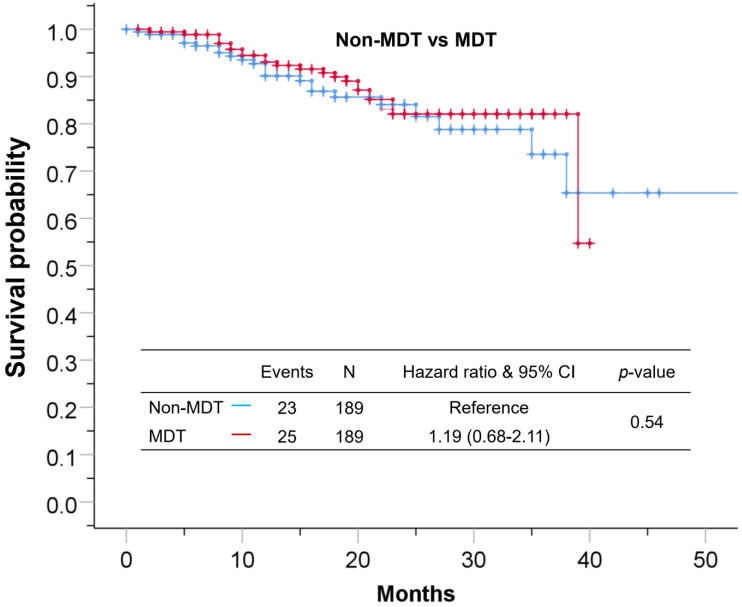
The Kaplan-Meier curve showing no statistical difference between patients in the MDT group and patients in the non-MDT group.

**Figure 3:**
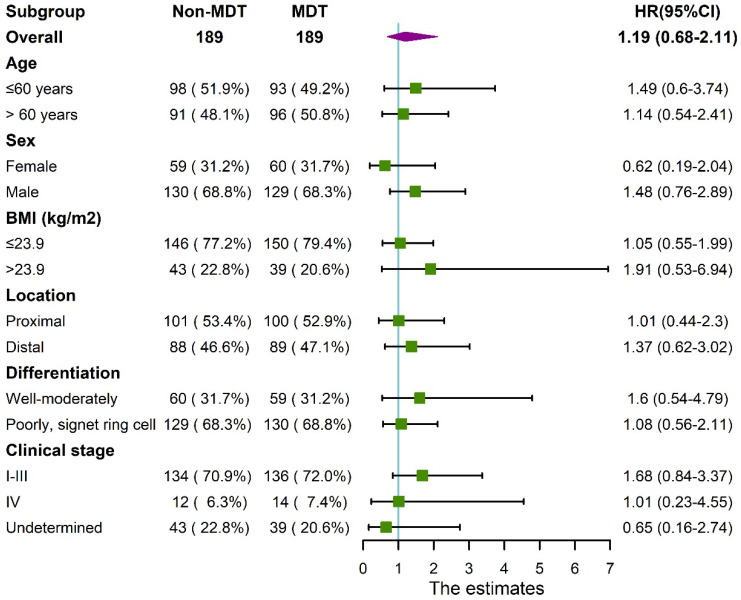
Forest plot showing no significant benefit from MDT consultation for patients in different subgroups.

Median follow-up time for the cohort was 17±11 months. The 1-year and 3-year overall survival rate for the total cohort was 92% and 66%, 90%, 62% for the non-MDT group, 93%, 68% for the MDT group. As shown in **Figure 2**, the overall survival was comparable between the MDT and non-MDT groups (p=0.54). In subgroup survival analysis (**Figure 3**), MDT groups also failed to show any superiority compared to the non-MDT group. Therefore, these data suggested that MDT consultation has limited effects on improving the survival of the patients.

## Discussion

Although the MDT approach has been proposed in cancer treatments, especially for individualized therapies, its effectiveness remains controversial due to the lack of strong evidence demonstrating its advantages in improving clinical outcomes 13, 14. This single-center retrospective study suggested that MDT consultation has limited effectiveness in improving staging accuracy and treatment outcomes including overall survival for patients with resectable gastric cancer. Moreover, the data indicated that patient compliance may affect the effectiveness of MDT consultation.

One important purpose of MDT is to improve staging accuracy of cancer patients. However, contrary to the previous study 8, our results showed that the accuracy of TNM staging was not improved by MDT consultation even after excluding patients who received preoperative chemotherapy that may ultimately alter the final pathological stage of tumors. One possible reason is the poor patient compliance that leads to insufficient examinations. To improve staging accuracy, multi-modality imaging techniques must be applied. For example, endoscopic ultrasonography has been proven to be the most reliable and accurate technique for T staging 15-17, PET-CT and MRI are important approaches for differentiating suspicious metastatic site 18-21. In our study cohort, these examinations were frequently recommended by the MDT but rarely accepted by the patients due to the worry of further delay of surgery and expensive cost.

Regarding peritoneum metastasis, staging laparoscopy has been recommended by several studies 22-24. Although our data showed that the recommendation of exploratory laparoscopy was accepted by the majority of patients, it was only accepted as part of the laparoscopic gastrectomy surgery; and yet none of the exploratory laparoscopies were performed simply as a method of staging inspection without immediate subsequent resection. Thus, the clinical stages of the MDT group were still mostly established on routine contrast-enhanced CT imaging, which limited the room for improvement, even with the reassessment by the MDT.

As for the treatment plan, all patients at the early stage (clinical stage I) were recommended with direct resection surgery by the MDT. Endoscopic submucosal dissection was only applicable for patients with tumor invasion depth under T1a and with no evidence of lymph node metastasis 25. Also, the probability of insidious lymph node metastasis in early gastric cancer is not negligible 26. Hence, endoscopic resection was rarely recommended. For patients at clinical stage II-III, there was a controversy on the necessity of neoadjuvant chemotherapy. The Chinese gastric cancer diagnosis and treatment guideline recommended it only for cT3/4N+ patients^11^, but it also stated that the survival benefit was still uncertain and well-designed trials were lacking 27, 28, the guideline encouraged MDT to develop individualized strategy based on the tumor feature and the patients' condition. The Japanese guideline recommend neoadjuvant chemotherapy for cT2-4 patients with evident of bulky lymph nodes metastasis 29. Thus, our MDT had tried to balance the differences across guidelines and attempt to develop more personalized treatment strategies for this subgroup. Overall speaking, the MDT tends to recommend neoadjuvant chemotherapy to patients with good performance status, present of adjacent organ invasion or bulky lymph node, and direct surgical resection otherwise. As a result, patients in the MDT group had received more preoperative chemotherapy, applied regimens mainly include mFLOT 28 and SOX 30, but the achievement of R0 resection and survival was not improved. For patients at clinical stage IV, the treatment recommendations were vastly different due to the great heterogeneous nature of the disease. Surgery could only be attempted when curative resection is achievable or in case of obstruction or bleeding 29, but in most case, systemic chemotherapy was the first choice. In this study, all patients enrolled had already received gastrectomy. But preoperative MDT consultation failed to show any positive impact on the survival of this subgroup. The MDT had also recommended a variety of treatments and supportive care that may have been beneficial to these patients 31, but regrettably, they were rarely accepted by the patients. As for radiotherapy 32, the National Comprehensive Cancer Network guideline for gastric cancer recommends it as part of perioperative therapy, but in the guidelines of Asia, it is recommended only in patients with unresectable metastatic sites 29, 33. In our study, less than 10% of patients were recommended to receive radiotherapy, but none of them complied.

Regarding survival benefits, MDT consultation did not improve overall survival rate in our study. In the subgroup analysis, the MDT group did not show any survival advantage for patients with tumors in different locations, differentiation, or different clinical stages, as shown in the forest plot in **Figure 3**. Possible explanations could be as followed. First, the patients' poor compliance with further examinations weakened the effectiveness of MDT consultation. Moreover, most of treatment plans recommended for patients at advanced stages were invalidated strategies, such as neoadjuvant chemotherapy and radiotherapy, which were controversial in term of their survival benefit. Besides, the recommended approaches by the MDT for patients at early stages were mostly standardized plans, which was not much different from the non-MDT group. These factors could notably limit the room for survival improvement of MDT consultation. Thus, we believe that patient compliance and staging accuracy are key factors affecting the survival of patients with gastric cancer.

Noticeably, several limitations to this study need to be acknowledged. First, selection bias cannot be neglected. All patients enrolled in this study eventually underwent gastrectomy, those who refused surgery or lost the opportunity to undergone surgery due to progression during chemotherapy were not included, which may result in selection bias. Second, the number of studied populations distributed unevenly among the various stages. In particular, most of the enrolled patients were at stage III; and the sample size of stage I, II, and IV may not be sufficient to testify the effectiveness of MDT. Third, the follow-up time for this cohort was relatively short, which could weaken the statistical significance of survival analysis. Despite these limitations, this is the first study exploring the effectiveness of MDT consultation on patients with resectable gastric cancer with detailed data provided. The utility of propensity score matching method allows us to imitate randomized inclusion and compare the effect of MDT between the two groups, making the results more persuasive.

## Conclusion

This study demonstrates that MDT consultation has limited effect in improving the staging accuracy and treatment outcomes including the survival benefit for patients with resectable gastric cancer, likely due to insufficient examination and poor patient compliance. To improve the effectiveness of MDT consultation, further efforts should be made to improve staging accuracy and the compliance of patients.

## Figures and Tables

**Table 1:** Patient characteristics including the baseline information and p-value of univariate analysis

	Non-MDT (n=189)	MDT (n=189)	Total (n=378)	*P*-value
**Gender**				
Female	59 (31.2%)	60 (31.7%)	119 (31.5%)	1
Male	130 (68.8%)	129 (68.3%)	259 (68.5%)	
Age (years)	58±12	59±12	58±12	0.61
BMI (kg/m^2^)	21.7±3.6	21.8±2.9	21.7±3.3	0.69
**Tumor location**				
Esophagogastric junction	15 (7.9%)	26 (13.8%)	41 (10.8%)	0.1
Upper third	38 (20.1%)	41 (21.7%)	79 (20.9%)	
Middle third	47 (24.9%)	30 (15.9%)	77 (20.4%)	
Lower third	88 (46.6%)	89 (47.1%)	177 (46.8%)	
Whole stomach	1 (0.5%)	3 (1.6%)	4 (1.1%)	
**Tumor histological classification**				
Well differentiated adenocarcinoma	9 (4.8%)	7 (3.7%)	16 (4.2%)	0.71
Moderately differentiated adenocarcinoma	51 (27.0%)	52 (27.5%)	103 (27.2%)	
Poorly differentiated adenocarcinoma	116 (61.4%)	114 (60.3%)	230 (60.8%)	
Signet ring cell	3 (1.6%)	8 (4.2%)	11 (2.9%)	
Mucous adenocarcinoma	8 (4.2%)	7 (3.7%)	15 (4.0%)	
Papillary carcinoma	2 (1.1%)	1 (0.5%)	3 (0.8%)	
**Clinical stage**				
I	15 (7.9%)	11 (5.8%)	26 (6.9%)	0.32
II	3 (1.6%)	10 (5.3%)	13 (3.4%)	
III	116 (61.4%)	115 (60.8%)	231 (61.1%)	
IV	12 (6.3%)	14 (7.4%)	26 (6.9%)	
Undetermined	43 (22.8%)	39 (20.6%)	82 (21.7%)	
**Preoperative chemotherapy**				
No	136 (72.0%)	115 (60.8%)	251 (66.4%)	0.03
Yes	53 (28.0%)	74 (39.2%)	127 (33.6%)	
**Preoperative chemotherapy regimen (n=127)**				
mFLOT	31 (58.5%)	44 (59.5%)	75 (59.1%)	0.45
SOX, FOLFOX or XELOX	16 (30.2%)	26 (35.1%)	42 (33.1%)	
Other	6 (11.3%)	4 (5.4%)	10 (7.9%)	
**Resection approach**				
Open	53 (28.0%)	33 (17.5%)	86 (22.8%)	0.02
Laparoscopic	136 (72.0%)	156 (82.5%)	292 (77.2%)	
**Resection extend**				
Distal gastrectomy	94 (49.7%)	95 (50.3%)	189 (50.0%)	0.93
Proximal gastrectomy	4 (2.1%)	5 (2.6%)	9 (2.4%)	
Total gastrectomy	91 (48.1%)	89 (47.1%)	180 (47.6%)	
**Completeness of resection**				
R0	168 (88.9%)	177 (93.7%)	345 (91.3%)	0.73
R1 or R2	21 (11.1%)	12 (6.3%)	17 (8.7%)	
Length of hospitalization (days)	14±9	14±9	14±9	0.44
Number of Harvested lymph nodes	32±15	29±12	31±14	0.01
**Vascular tumor embolus**				
No	137 (72.5%)	132 (69.8%)	269 (71.2%)	0.65
Yes	52 (27.5%)	57 (30.2%)	109 (28.8%)	
**Nerve invasion**				
No	112 (59.3%)	118 (62.4%)	230 (60.8%)	0.6
Yes	77 (40.7%)	71 (37.6%)	148 (39.2%)	
**Pathological stage**				0.09
T0N0M0	8 (4.8%)	9 (5.0%)	17 (4.9%)	
IA	14 (8.4%)	17 (9.5%)	31 (9.0%)	
IB	10 (6.0%)	16 (8.9%)	26 (7.5%)	
IIA	30 (18.0%)	45 (25.1%)	75 (21.7%)	
IIB	46 (27.5%)	28 (15.6%)	74 (21.4%)	
IIIA	21 (12.6%)	32 (17.9%)	53 (15.3%)	
IIIC	20 (12.0%)	14 (7.8%)	34 (9.8%)	
IV	13 (7.8%)	16 (8.9%)	29 (8.4%)	
Undetermined	5 (3.0%)	2 (1.1%)	7 (2.0%)	

**Abbreviations**: MDT: Multidisciplinary team consultation; BMI: Body mass index; mFLOT: Docetaxel 50~60 mg/m^2^ + Oxaliplatin 85 mg/m^2^ + Fluorouracil 2800 mg/m^2^ iv over 48 hours; every 2 weeks. SOX: Oxaliplatin 130 mg/m^2^ iv + Tegafur Gimeracil Oteracil Potassium Capsule 40~60 mg bid D1-D14; every 3 weeks. XELOX: Oxaliplatin 130 mg/m^2^ + Capecitabine 1000mg/m^2^ bid D1-D14; every 3 weeks. FOLFOX: Oxaliplatin 85 mg/m^2^ + Fluorouracil 2800 mg/m^2^ civ over 48 hours; every 2 weeks. The dosage of the regimens listed above might be slightly modified according to the preference of the oncologist.

**Table 2:**
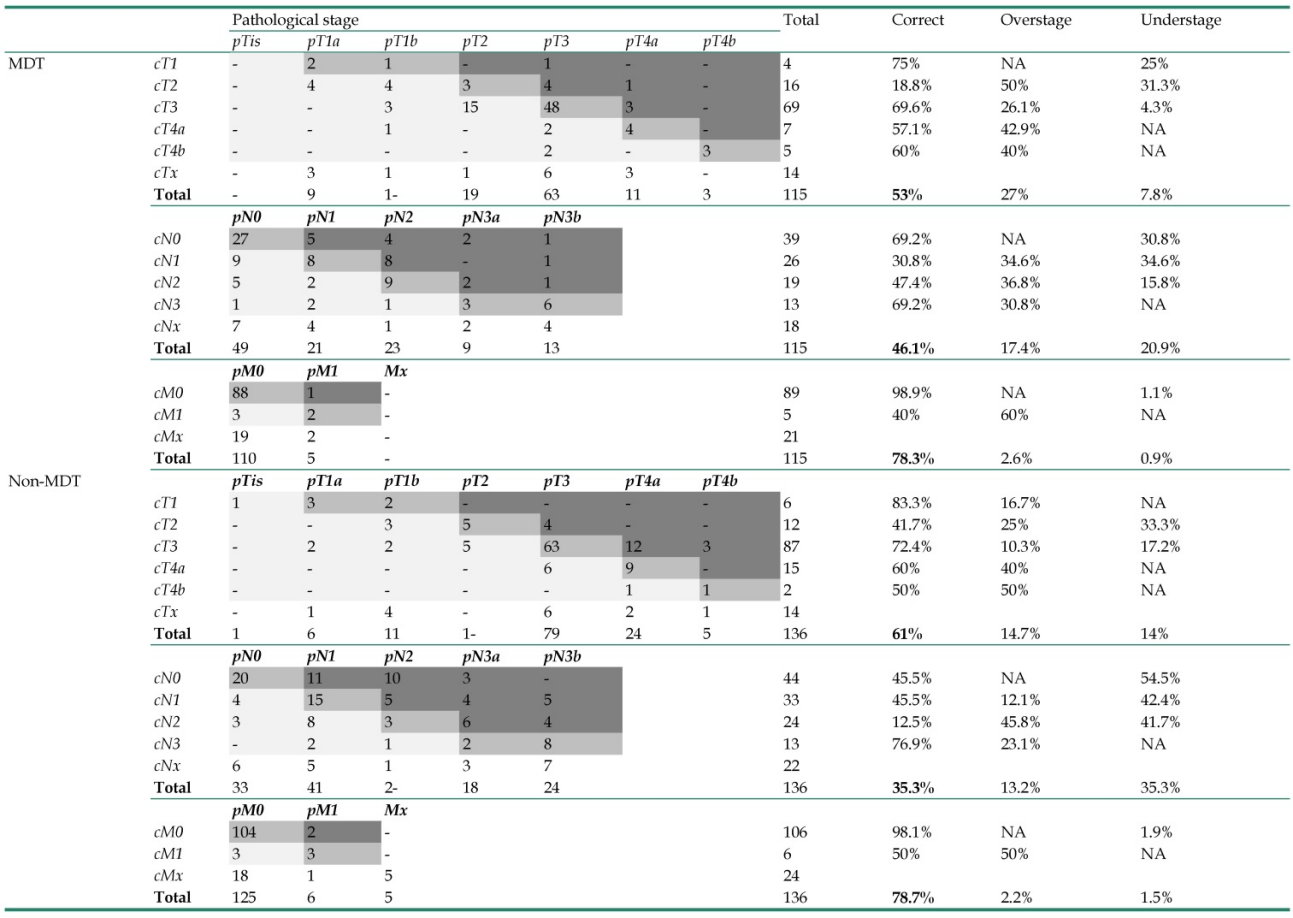
Staging accuracy of the MDT group and the non-MDT group (n=278, patients with neoadjuvant chemotherapy excluded)

**Table 3:** Recommendations of the MDT and the corresponding patient compliance

Clinical stage	Pre-operative chemotherapy	Direct tumor resection	Further investigation	Exploratory laparoscopy	Radiotherapy	Palliative therapy
I (n=11)	0	11 (100%)	4 (36%)	0	0	0
II (n=10)	3 (30%)	7 (70%)	1 (10%)	1 (10%)	1 (10%)	0
III (n=115)	45 (39%)	48 (42%)	19 (17%)	13 (11%)	4 (3%)	0
IV (n=14)	6 (43%)	2 (14%)	2 (14%)	8 (57%)	3 (21%)	6 (43%)
Undetermined (n=39)	14 (36%)	1 (3%)	6 (15%)	22 (56%)	3 (8%)	1 (3%)
Total (n=189)	68 (36%)	84 (44%)	32 (17%)	29 (15%)	11 (6%)	7 (4%)
Compliance	44/68, 65%	68/84, 81%	11/32, 34%	24/29, 83%	0	0
